# Hepatitis B (HBsAg) prevalence among obstetric patients in Caluquembe, Angola, 2023–2024

**DOI:** 10.1371/journal.pone.0327426

**Published:** 2025-07-03

**Authors:** Anna E. Eberwein, Priscila Ribeiro Cummings, Daniel Cummings, Julia Andre, Kathryn H. Jacobsen

**Affiliations:** 1 Hospital Evangélico de Caluquembe, Caluquembe, Huíla, Angola; 2 Department of Health Studies, University of Richmond, Richmond, Virginia, United States of America; Kwame Nkrumah University of Science and Technology, GHANA

## Abstract

**Objectives:**

Newborns who contract hepatitis B virus (HBV) infections at birth often develop chronic infections that can cause cirrhosis, liver cancer, and death in middle adulthood. Birth doses of hepatitis B vaccine can be lifesaving for babies born to mothers with hepatitis B infections. We aimed to measure hepatitis B prevalence among maternity patients in Huíla, a rural province in southwestern Angola.

**Methods:**

We conducted a prospective case series study among 317 peripartum women at the Hospital Evangélico de Caluquembe from November 2023 to February 2024. Each participant received a point-of-care hepatitis B surface antigen (HBsAg) test and was asked about HBV and vaccine knowledge. We also conducted qualitative interviews about HBV prevention with 26 healthcare workers.

**Results:**

The HBsAg prevalence was 4.7%. None of the women who tested positive was previously aware of her status. Only about one-third of the women expressed familiarity with hepatitis B or HBV vaccines, and almost none reported that their older children had received HBV vaccines. Maternal health workers proposed hosting community meetings to provide education about HBV and birth-dose vaccination.

**Conclusions:**

Only about half of Angolan babies are born at healthcare facilities, but more than 80% of women attend at least one antenatal care visit. Improved access to and uptake of hepatitis B screening at antenatal checkups is essential for ensuring that babies born to women with chronic hepatitis B infections are able to receive birth dose hepatitis B vaccines.

## Introduction

Up to 90% of the newborns who contract hepatitis B virus (HBV) through vertical (mother-to-child) transmission who do not receive hepatitis B vaccination shortly after birth will develop a chronic infection that significantly increases their lifetime risk of liver cirrhosis and hepatocellular carcinoma [1]. About two-thirds of new cases of HBV infection occur in Africa, yet fewer than one-fifth of newborns in the region receive the hepatitis B vaccine within 24 hours of birth [2].

Although the Angolan Ministry of Health added a birth dose of hepatitis B vaccine to its routine childhood vaccine schedule in 2015 [3], recommending a first dose of vaccine within 24 hours of birth and then boosters at ages 2, 4, and 6 months, very few Angolan infants receive a birth dose of hepatitis B vaccine and only about half receive at least three total doses [4]. Increasing the proportion of newborns who receive birth doses of hepatitis B vaccine to prevent vertical transmission is a priority for the many groups working to reduce the burden from acute and chronic viral hepatitis, including international groups like the World Health Organization (WHO) and the Coalition for Global Hepatitis Elimination (CGHE) [2,5]. Identifying under-vaccinated populations is an important first step toward improving vaccine delivery services.

Since babies born to mothers with HBV infections are at high risk of contracting the virus, serosurveys of maternal hepatitis B status are an important tool for identifying high-risk infant populations. Hepatitis B surface antigen (HBsAg) tests are qualitative immunoassays that provide evidence of active hepatitis B infection, and they are recommended by the WHO as the best test for diagnosing chronic HBV infections [6]. Most of the previous studies of HBsAg prevalence in Angola were conducted in the capital region, Luanda, including studies of women attending antenatal care clinics in 2016–2017 (n = 878, 25.7%) [7]; blood donors in 2005–2010 (n = 8043, 12.5%) and 2011–2016 (n = 2734, 50.2%) [8,9]; patients seeking HIV testing in the early 2000s (n = 431, 9.3%) [10]; and hospital staff, visitors, and patients in 2007 (n = 508, 15.1%) [11] and the early 2000s (n = 1103, 13%) [12]. These urban studies are not necessarily representative of the epidemiological situation in other parts of the country. National surveillance studies in 2000–2004 (n = 78,000, 8.7%) and 2010–2011 (n = 78,275, 6.7%) suggested decreasing HBV prevalence rates [13], but to our knowledge no nationally-representative serosurveys have been conducted in the past decade.

We aimed to measure HBV prevalence among peripartum inpatients at a hospital in Huíla province, in southwestern Angola, so that we could understand the current prevalence of HBsAg among pregnant women and the need for birth-dose vaccination in this rural area far from the capital city.

## Materials and methods

We conducted a prospective case series study of hepatitis B among women admitted to the maternity ward at Hospital Evangélico de Caluquembe between 13 November 2023 and 9 February 2024. Women admitted to the maternity ward who were pregnant or within six weeks of delivery were eligible to be included in the study. Women in the labor and delivery room and those who were experiencing delirium were not approached by study personnel. In total, more than 96% of all women admitted to the ward were invited to participate in the study and 99% of the invited women consented to participation and completed a brief interview with study personnel. The questionnaire used for the interview was developed in consultation with local women’s healthcare providers. Most interviews were conducted in Portuguese, but patients could opt to be interviewed in Umbundu.

Prior to the initiation of data collection, the Ethics Review Committee of Hospital Evangélico de Caluquembe convened to review the research protocol. The committee determined that the research project was not experimental research and there were no concerns about the plans for recruiting, informed consent, or data collection, storage and analysis. A letter of approval was provided by the hospital director per the group’s operating guidelines. Potential participants were provided with information about the study goals and procedures. The consent process emphasized that participation was voluntary and that all women on the ward would receive the same quality of care and the same voluntary hepatitis B test regardless of their decision about whether to participate in the study. Consent was obtained verbally from patients and was witnessed by a staff person who was not a member of the research team. To ensure the anonymity of participants and the confidentiality of their responses, the study database did not include any identifiable patient information.

Interviews were conducted daily after hospital rounds. The questionnaire used for the interviews included items about familiarity with HBV and the hepatitis B vaccine; reproductive history (total number of pregnancies, total number of stillbirths, and total number of live births); the current or most recent pregnancy, including the due date (if still pregnant) or birth date (if the baby had already been delivered), the number of prenatal checkups, the types of pregnancy complications experienced, and the type of delivery (such as whether a Cesarean section was done), and the outcome; and a few questions about the number of living children, the vaccination status of those children, and the hepatitis B vaccination status of the neonate. All of these questions could be answered with single-word responses, such as a yes, a no, or a number, except for the question about the vaccines older children received, for which several words of response might be appropriate. We considered all answers that were appropriate for the question (such as all “yes” or “no” responses to a yes/no question) to be valid. Since most of the participants received antenatal and other primary care services at rural clinics rather than the hospital, and their records were therefore not available to the research team, we did not attempt to validate the responses with chart reviews.

Blood samples were collected via venipuncture for blood serum tests or finger prick for whole blood (WB) tests. MeriScreen HBsAg blood serum or WB tests (Meril Diagnostics; sensitivity >98%, specificity >99.5%, as per the manufacturer) or Bioline HBsAg WB serum, plasma, or WB tests (Abbott; sensitivity 100%, specificity 100%, as per the manufacturer) were conducted by trained laboratory technicians according to manufacturer instructions. None of the test kits were past their expiration dates. All tested patients received the results of their hepatitis B tests prior to being discharged from the hospital. Patients with positive test results received counseling that encouraged them to undergo annual testing for liver disease, notify their partners about their hepatitis B status, and make sure that current and future children are tested for hepatitis B and vaccinated against the disease, including future children receiving birth doses of hepatitis B vaccine. (Treatment for hepatitis B is not currently available at Hospital Evangélico de Caluquembe, and only adults with HIV are eligible to be treated for hepatitis B at the district hospital).

We used Epi Info to analyze the distribution of responses to questionnaire items and to examine associations between questionnaire responses and laboratory test results. Associations were tested using chi-squared tests (with a significance level of α = 0.05) and odds ratios (with 95% confidence intervals).

In response to the CGHE call for qualitative research about opportunities to improve interventions for reducing mother-to-child transmission of HBV, including studies about community education and communication tools [5], we also conducted brief interviews during March 2024 with 26 of the clinical staff in the hospital’s maternity ward. Interviewees were recruited based on their availability during that month. We asked the participating healthcare workers for practice-based insights about the interventions that might be useful for increasing knowledge about HBV and hepatitis B vaccinations, increasing the number of women who are tested for HBsAg no matter where they seek antenatal care and deliver their babies, and increasing hospital birth rates and vaccination rates. Our qualitative analysis of their responses summarized common keywords and themes.

## Results

In total, 317 of the 328 volunteer patients had test results that met the manufacturer’s standards for validity. Fifteen (4.7%) of the 317 valid HBsAg tests were positive. None of the 15 HBsAg-positive women were aware that they had hepatitis B infections. There were no differences in HBsAg seropositivity or awareness of HBV and hepatitis B vaccines by age group, reproductive history, or the proximity of the home to the hospital. Among the 191 participants with living children aged 1 year or older, those who had previously heard about HBV were slightly more likely to report that their children had received at least one vaccine of any type (51/67 = 76%) than those who said they had never heard about HBV before (81/124 = 65%, p = 0.13). However, only 9 of those 191 women reported having a child aged 1 year or older who had been vaccinated against hepatitis B.

The staff on the maternity ward expressed strong agreement that health education about prenatal HBV testing and birth-dose hepatitis B vaccines for newborns would help increase use of these services (25/26 = 96%). When asked an open-ended question about the most effective strategies for providing education about hepatitis B, most staff mentioned community-based presentations as a useful communication tool (24/26 = 92%). Community-based presentations involve inviting a community to gather in a church or field to listen to a public health worker or clinical healthcare worker verbally present on a topic. These types of community gatherings have been used for public education about other health issues in the past.

## Discussion

Our 4.7% HBsAg prevalence rate is lower than the rates found in a similar study of maternity ward patients in Lubango, also in Huíla province, in 2016–2017 (n = 500, 8.6%); in a study of pregnant women in Kuito, in Bié province in central Angola, in 2007 (n = 1012, 8.7%); and a study of adults in Mancusso, in Cuando province in southern Angola, in 1992 (n = 201, 13.3%) [14–16]. Our results are consistent with the approximately 5% prevalence rate of hepatitis B infection in the African region [2]. It is possible that the prevalence of hepatitis B infection is lower in Angola now than it was when those other studies were conducted. If so, that trend would be consistent with decreases in HBV prevalence observed in other parts of the African region [17]. However, an approximately 5% prevalence rate is still high by WHO standards.

The WHO recommends that all adults in places with a 2% or greater HBsAg seroprevalence rate be offered routine testing and that all pregnant women in these areas receive point-of-care screening for HBsAg as part of routine antenatal clinic services [2]. It is concerning that none of the women who tested positive for hepatitis B in our study knew their status prior to participating in our study. The WHO and GCHE aim by 2030 for at least 90% of people with chronic hepatitis B to be diagnosed so that at least 80% of individuals with chronic HBV infection can treated [2,5]. In Angola, the hepatitis B case detection rate is very low and treatment remains unavailable in many areas, especially in rural parts of the country.

The WHO also calls for at least 90% of newborns to receive timely birth doses of hepatitis B vaccine to prevent vertical transmission of HBV [2]. Given that about half of pregnant women in Angola do not give birth at a healthcare facility [18], universal vaccination of newborns against HBV is not a realistic target for 2030. An intermediate step toward achieving the goal of universal vaccination would be to set a goal of testing 100% of pregnant women for hepatitis B during antenatal visits, and to encourage all women who test positive for HBsAg to deliver their babies at healthcare facilities where birth dose hepatitis B vaccinations are available. More than 80% of pregnant women in Angola have at least one antenatal visit and more than 60% have at least four visits [18], so scaled up testing at antenatal clinics could enable robust progress toward vaccinating at least 90% of high-risk neonates in Angola by 2030.

It is reasonable to assume that many rural areas in African countries face challenges related to increasing awareness of hepatitis B, testing for chronic hepatitis B infections, and delivery of birth dose hepatitis B vaccines to neonates whose mothers have hepatitis B that are similar to the ones we observed in Angola. Global goals for increasing testing, diagnosis, treatment, and vaccination will not be achieved without greater attention being placed on expanding access to these services in rural areas.

## Supporting information

S1 Data Data file. (XLSX)

S2 File Manuscript in Portuguese. (PDF)

S3 File Abstract in Spanish. (PDF)

S4 File Abstract in French. (PDF)
