# Supplementary material for: Hepatitis B (HBsAg) prevalence among obstetric patients in Caluquembe, Angola, 2023–2024
Source: PLoS One. 2025 Jul 3;20(7):e0327426. doi: 10.1371/journal.pone.0327426 (PMC12225797; doi:10.1371/journal.pone.0327426)
Supplement: S2 File — (PDF) [file pone.0327426.s002.pdf]

Resumo em Português

Prevalência de hepatite B (HBsAg) entre pacientes obstétricas em Caluquembe, Angola, 2023–2024

Objectivos: Os recém-nascidos que contraem infecções pelo vírus da hepatite B (VHB) ao nascer desenvolvem frequentemente infecções crónicas que podem causar cirrose, cancro do fígado e morte na meia idade adulta. Doses da vacina contra hepatite B ao nascer podem salvar a vida de bebês nascidos de mães com hepatite B. O nosso objectivo foi medir a prevalência do VHB entre pacientes grávidas na Huíla, um distrito rural no sudoeste de Angola.

Métodos: Realizamos um estudo prospectivo de série de casos com 317 mulheres periparto do Hospital Evangélico de Kalukembe de novembro de 2023 a fevereiro de 2024. Cada participante recebeu um teste no local de atendimento de antígeno de superfície da hepatite B (HBsAg) e foi questionado sobre seu conhecimento sobre o HBV e a vacina. Também realizamos entrevistas qualitativas sobre prevenção do VHB com 26 profissionais de saúde.

Resultados: A prevalência de HBsAg foi de 4,7%. Nenhuma das mulheres que testaram positivo tinha conhecimento prévio do seu estado. Apenas cerca de um terço das mulheres estavam familiarizadas com o VHB ou vacinas contra hepatite B, e quase nenhum dos participantes relatou que seus filhos mais velhos haviam recebido vacinas contra VHB. Os profissionais de saúde materna propuseram reuniões comunitárias para fornecer educação sobre o VHB e a vacinação com doses à nascença.

Conclusões: Apenas cerca de metade dos bebés angolanos nascem em centros de saúde, mas mais de 80% das mulheres comparecem a pelo menos uma consulta pré-natal. Melhorar o acesso e a adesão ao rastreio da hepatite B durante o pré-natal é essencial para garantir que bebês

nascidos de mulheres com infeções crónicas por hepatite B possam receber a dose de vacinação correspondente à dose de vacinação ao nascer.

Palavras-chave: hepatite B; transmissão vertical; Angola; VHB; vacinas; saúde perinatal

Título abreviado: HBsAg em Caluquembe, Angola

Manuscrito em Português

Prevalência de hepatite B (HBsAg) entre pacientes obstétricas em Caluquembe, Angola, 2023–2024

Introdução

Até 90% dos recém-nascidos que contraem o vírus da hepatite B (HBV) por transmissão vertical (da mãe para filho) e não recebem a vacinação contra hepatite B logo após o nascimento desenvolverão uma infecção crônica que aumenta significativamente o risco ao longo da vida de cirrose hepática e carcinoma hepatocelular.[1] Cerca de dois terços dos novos casos de infecção por VHB ocorrem na África, em parte porque menos de um quinto dos recém-nascidos na região recebem a vacina contra a hepatite B.[2]

O Ministério da Saúde de Angola tem adicionado uma dose da vacina contra hepatite B ao nascimento ao seu calendário de imunização infantil de rotina em 2015,[3] recomendando uma primeira dose da vacina dentro de 24 horas após o nascimento e, em seguida, reforços aos 2, 4 e 6 meses de idade. Infelizmente, muito poucos bebês angolanos recebem uma dose da vacina contra hepatite B ao nascer, e apenas cerca de metade recebe pelo menos três doses.[4] Aumentar a proporção de recém-nascidos que recebem doses da vacina contra hepatite B ao nascer para prevenir a transmissão vertical é uma prioridade para os muitos grupos que trabalham para reduzir a carga da hepatite viral aguda e crônica, incluindo grupos internacionais como a Organização Mundial da Saúde (OMS) e a Coalizão para Eliminação Global da Hepatite (CGHE).[2,5] Identificar populações subvacinadas é um primeiro passo importante para melhorar os serviços de distribuição de vacinas.

Como bebês nascidos de mães com infecção por HBV apresentam alto risco de adquirir o vírus, os exames sorológicos do estado materno de hepatite B são uma ferramenta importante

para identificar populações de bebês de alto risco. Os testes de antígeno de superfície da hepatite B (HBsAg) são imunoensaios qualitativos que fornecem evidências de infecção ativa por hepatite B e são recomendados pela OMS como o melhor teste para o diagnóstico de infecções crônicas por HBV.[6] A maioria dos estudos anteriores sobre a prevalência do HBsAg em Angola foram conduzida na região da capital, Luanda, incluindo estudos de mulheres que frequentaram clínicas pré-natais em 2016–2017 (n = 466, 8,6%);[7] doadores de sangue em 2005–2010 (n = 8043, 12,5%) e 2011–2016 (n = 2734, 50,2%);[8,9] pacientes que procuraram testes de HIV em 2010–2012 (n = 431, 9,3%);[10] e funcionários, visitantes e pacientes de hospitais em 2007 (n = 508, 15,1%)[11] e início dos anos 2000 (n = 1103, 13%).[12] Esses estudos urbanos não são necessariamente representativos da situação epidemiológica em outras partes do país. Estudos nacionais de vigilância em 2000-2004 (n = 78.000, 8,7%) e 2010-2011 (n = 78.275, 6,7%) sugeriram taxas de prevalência decrescentes de VHB,[13] mas, até onde sabemos, nenhum inquérito sorológico nacionalmente representativo foi realizado na última década.

O nosso objectivo foi medir a prevalência do HBV entre pacientes periparto internados num hospital na província da Huíla, no sudoeste de Angola, para que pudéssemos compreender a prevalência actual do HBsAg entre mulheres grávidas e a necessidade de vacinação com dose à nascença nesta zona rural longe da capital.

Métodos

Realizamos um estudo prospectivo de série de casos de hepatite B entre mulheres internadas na maternidade do Hospital Evangélico de Caluquembe entre 13 de novembro de 2023 e 9 de fevereiro de 2024. Mulheres internadas na maternidade que estavam grávidas ou dentro de seis semanas após o parto eram elegíveis para serem incluídas no estudo. As mulheres

na sala de parto e aquelas que estavam visivelmente doentes não foram abordadas pela equipe do estudo. No total, mais de 96% de todas as mulheres internadas na enfermaria foram convidadas a participar do estudo, e 99% das mulheres convidadas consentiram em participar e completaram uma breve entrevista com a equipe do estudo. O questionário utilizado para a entrevista foi desenvolvido em consulta com os prestadores locais de cuidados de saúde da mulher. A maioria das entrevistas foram realizadas em português, mas os pacientes podiam optar por serem entrevistados em umbundo.

Antes do início da coleta de dados, o Comitê de Ética em Pesquisa do Hospital Evangélico de Caluquembe se reuniu para revisar o protocolo de pesquisa. O comitê determinou que o projeto de pesquisa não era uma pesquisa experimental e não havia preocupações sobre os planos de recrutamento, consentimento informado ou coleta, armazenamento e análise de dados. Uma carta de aprovação foi fornecida pelo administrador do hospital de acordo com as diretrizes operacionais do grupo. Os potencias participantes receberam informação sobre os objetivos e procedimentos do estudo. O processo de consentimento enfatizou que a participação era voluntária e que todas as mulheres na enfermaria receberiam a mesma qualidade de atendimento e o mesmo teste voluntário de hepatite B, independentemente de sua decisão de participar do estudo. O consentimento foi obtido verbalmente dos pacientes e testemunhado por um membro da equipe que não era membro da equipe de pesquisa. Para garantir o anonimato dos participantes e a confidencialidade de suas respostas, o banco de dados do estudo não incluiu informação identificável do paciente.

As entrevistas foram realizadas diariamente após as rondas hospitalares. O questionário utilizado para as entrevistas incluiu itens sobre familiaridade com a vacina contra o VHB e a hepatite B; histórico reprodutivo (número total de gestações, número total de nados mortos e

número total de nascidos vivos); gravidez atual ou mais recente, incluindo data prevista para o parto (se ainda grávida) ou data de nascimento (se o bebê já tivesse nascido), número de consultas pré-natais, tipos de complicações vivenciadas na gravidez, e modos de parto (como se foi realizada uma cesárea) e desfecho; e algumas perguntas sobre o número de nascidos vivos, o estado de vacinação dessas crianças e o estado de vacinação contra a hepatite B do recém-nascido. Todas essas perguntas podiam ser respondidas com uma única palavra, como sim, não ou um número, exceto a pergunta sobre as vacinas recebidas por crianças mais velhas, para a qual respostas com múltiplas palavras poderiam ser apropriadas. Consideramos todas as respostas que eram apropriadas para a pergunta (como todas as respostas "sim" ou "não" a uma pergunta sim/não) como válidas. Como a maioria dos participantes receberam serviços pré-natais e outros serviços de atenção primária em clínicas rurais em vez de hospitais, e seus registros não estavam disponíveis para a equipe de pesquisa, não tentamos validar as respostas com revisões de gráficos.

Amostras de sangue foram coletadas por punção venosa para exames de soro sanguíneo ou picada no dedo para exames de sangue total. MeriScreen soro sanguíneo HBsAg ou exames de sangue total (Meril Diagnostics; sensibilidade > 98%, especificidade > 99,5%, dependendo do fabricante) ou Bioline HBsAg exames de soro sanguíneo, plasma ou sangue total (Abbott; sensibilidade 100%, especificidade 100%, de acordo com o fabricante) foram conduzidos por técnicos de laboratório treinados de acordo com as instruções do fabricante. Nenhum dos kits de teste expirou a data de validade. Todos os pacientes testados receberam resultados de testes de hepatite B antes de receberem alta hospitalar. Os pacientes com resultados de testes positivos receberam aconselhamento que os incentivou a realizar testes anuais para detectar doenças hepáticas, notificar os seus parceiros sobre o seu estado de hepatite B e garantir que as crianças

actuais e futuras sejam testadas para a hepatite B e vacinadas contra a doença, incluindo a administração ao nascer da vacina contra hepatite B para futuras crianças. (O tratamento para hepatite B não está atualmente disponível no Hospital Evangélico de Caluquembe, e apenas adultos com HIV são elegíveis para tratamento de hepatite B no hospital regional.)

Utilizamos o Epi Info para analisar a distribuição das respostas aos itens do questionário e examinar as associações entre as respostas e os resultados dos exames laboratoriais. As associações foram testadas por meio de testes qui-quadrado (com nível de significância de α = 0,05) e razões de chances (com intervalos de confiança de 95%).

Em resposta ao chamado do CGHE para pesquisa qualitativa sobre oportunidades de melhorar intervenções para reduzir a transmissão de HBV de mãe para filho, incluindo estudos sobre educação comunitária e ferramentas de comunicação,[5] também conduzimos breves entrevistas durante março de 2024 com 26 membros da equipe clínica da maternidade do hospital. Os entrevistados foram recrutados com base em sua disponibilidade durante aquele mês. Pedimos aos profissionais de saúde ideais baseados na prática sobre intervenções que poderiam ser úteis para aumentar o conhecimento sobre as vacinas contra HBV e hepatite B, aumentando o número de mulheres testadas para HBsAg, independentemente de onde elas buscam cuidados pré-natais e dão à luz seus bebês, e aumentando as taxas de natalidade hospitalar e as taxas de vacinação. Nossa análise qualitativa de suas respostas resumiu palavras-chave e temas comuns.

Resultados

Um total de 328 pacientes consentiram em participar do estudo, e 317 tiveram resultados de teste que atenderam aos padrões de validade do fabricante. Quinze (4,7%) dos 317 testes HBsAg válidos foram positivos. Nenhuma das 15 mulheres HBsAg-positivas sabia que tinha infecções por hepatite B. Não houve diferenças na soropositividade para HBsAg ou no conhecimento das vacinas contra HBV e hepatite B por faixa etária, histórico reprodutivo ou proximidade da casa ao hospital. Entre os 199 participantes com filhos vivos com 1 ano ou mais, aqueles que já tinham ouvido falar de HBV eram ligeiramente mais propensos a relatar que seus filhos haviam recebido pelo menos uma vacina de qualquer tipo (51/67 = 76%) do que aqueles que disseram nunca ter ouvido falar de HBV (81/124 = 65%, p = 0,13). No entanto, apenas 9 dessas 199 mulheres relataram ter um filho com 1 ano ou mais que havia sido vacinado contra hepatite B.

A equipe da maternidade expressou forte concordância de que a educação em saúde sobre testes pré-natais para HBV e vacinas contra hepatite B em doses ao nascer para recém-nascidos ajudaria a aumentar o uso desses serviços (25/26 = 96%). Quando questionados sobre as estratégias mais eficazes para fornecer educação sobre hepatite B, a maioria da equipe mencionou apresentações comunitárias como uma ferramenta de comunicação útil (24/26 = 92%). Apresentações comunitárias envolvem convidar uma comunidade a se reunir em uma igreja ou campo para ouvir um profissional de saúde pública ou agente de saúde clínico apresentar verbalmente um tópico. Esses tipos de reuniões comunitárias já foram usados para educação pública sobre outras questões de saúde no passado.

Discussão

Nossa taxa de prevalência de HBsAg de 4,7% é menor do que as taxas encontradas em um estudo semelhante de pacientes de maternidade em Lubango, também na província de Huíla, em 2016-2017 (n = 466, 8,6%); um estudo de mulheres grávidas em Kuito, província do Bié, centro de Angola, em 2007 (n = 1012, 8,7%); e um estudo de adultos em Mancusso, província do Cuando, sul de Angola, em 1992 (n = 201, 13,3%).[14-16] Nossos resultados são consistentes com a taxa de prevalência de infecção por hepatite B de aproximadamente 5% na região africana.[2] É possível que a prevalência de infecção por hepatite B seja menor em Angola agora do que era quando esses outros estudos foram conduzidos. Se assim for, essa tendência seria consistente com reduções na prevalência de HBV observadas em outras partes da região africana.[17] No entanto, uma taxa de prevalência de aproximadamente 5% ainda é alta pelos padrões da OMS.

A OMS recomenda que todos os adultos em locais com taxa de soroprevalência de HBsAg de 2% ou mais sejam submetidos a testes de rotina e que todas as gestantes nesses locais recebam o teste de HBsAg no local de atendimento como parte dos serviços de rotina da clínica pré-natal.[2] É preocupante que nenhuma das mulheres com teste positivo para hepatite B em nosso estudo soubesse de seu status sorológico. A OMS e o GCHE visam diagnosticar pelo menos 90% das pessoas com hepatite B crônica até 2030, para que pelo menos 80% dos indivíduos com infecção crônica por HBV possam ser tratados.[2,5] Em Angola, a taxa de detecção de casos de hepatite B é muito baixa e o tratamento continua indisponível em muitas áreas, especialmente nas áreas rurais do país.

A OMS também exige que pelo menos 90% dos recém-nascidos recebam doses oportunas da vacina contra hepatite B para prevenir a transmissão vertical do VHB.[2] Dado que aproximadamente metade das mulheres grávidas em Angola não dão à luz em um centro de saude,[18] a vacinação universal de recém-nascidos contra o VHB não é uma meta realista para

2030. Um passo intermediário para atingir a meta de vacinação universal seria estabelecer umace meta de testar 100% das mulheres grávidas para hepatite B durante as consultas de pré-natal e encorajar todas as mulheres com teste positivo para HBsAg a darem à luz seus bebês em centros de saúde onde as vacinas contra hepatite B de dose de nascimento estejam disponíveis. Mais de 80% das mulheres grávidas em Angola têm pelo menos uma consulta de pré-natal e mais de 60% têm pelo menos quatro consultas,[18] portanto, ampliar os testes em clínicas de pré-natal pode permitir um progresso robusto para vacinar pelo menos 90% dos recém-nascidos de alto risco em Angola até 2030.

É razoável supor que muitas áreas rurais em países africanos enfrentem desafios na conscientização sobre a hepatite B, na testagem para infecções crônicas por hepatite B e na administração da vacina contra hepatite B ao nascer para recém-nascidos cujas mães têm hepatite B semelhante à observada em Angola. As metas globais para aumentar a testagem, o diagnóstico, o tratamento e a vacinação não serão alcançadas sem maior atenção à expansão do acesso a esses serviços nas áreas rurais.

Referências

1. Dionne-Odom J, Njei B, Tita ATN. Elimination of vertical transmission of hepatitis B in Africa: a review of available tools and new opportunities. Clin Ther. 2018;40:1255-1267. https://doi.org/10.1016/j.clinthera.2018.05.016
2. World Health Organization (WHO). Global Hepatitis Report 2024: Action for Access in Low- and Middle-Income Countries. Geneva: WHO; 2024. https://www.who.int/publications/i/item/9789240091672
3. Njuguna HN, Ward JW, Kabore J, Hiebert L, Jacques-Carroll L, Khetsuriani N, Tohme RA. Introduction of Hepatitis B Birth Dose Vaccination in Africa: A Toolkit for National Immunization Technical Advisory Groups 2022. Decatur GA: The Task Force for Global Health; 2022. https://www.globalhep.org/sites/default/files/content/page/files/2022-12/HepB-BD%20NITAG%20toolkit%20final%20version_12-16-22_0-FINAL.pdf
4. Angola: WHO and UNICEF Estimates of Immunization Coverage: 2023 Revision. Geneva: WHO; 2023. https://cdn.who.int/media/docs/default-source/country-profiles/immunization/2024-country-profiles/immunization-2024-ago.pdf

5. Coalition for Global Hepatitis Elimination (CGHE). An Operational Research Roadmap to Simplify Hepatitis B Care in Low- and Middle-Income Countries. Decatur GA: The Task Force for Global Health; 2023. https://www.globalhep.org/projects-research/operational-research-roadmap-simplify-hepatitis-b-care-low-and-middle-income
6. World Health Organization (WHO). Consolidated Strategic Information Guidelines for Viral Hepatitis: Planning and Tracking Progress Towards Elimination. Geneva: WHO; 2019. https://iris.who.int/bitstream/handle/10665/310912/9789241515191-eng.pdf
7. Vueba AN, Almendra R, Santana P, Faria C, do Céu Sousa M. Prevalence of HIV and hepatitis B virus among pregnant women in Luanda (Angola): geospatial distribution and its association with socio-demographic and clinical-obstetric determinants. Virol J. 2021;18:239. https://doi.org/10.1186/s12985-021-01698-7
8. Fernández Águila JD, Baptista Pocombo BL, Sequeira Pataca SA, Fragoso M, Rivero Jiménez CR. Donación de sangre y prevalencia de infecciones transmitidas por la sangre en una clínica de Luanda, Angola. MediSur. 2012;10:111-119. http://scielo.sld.cu/scielo.php?pid=S1727-897X2012000200006&script=sci_arttext
9. Quintas AE, Dias CC, Cogle ADC, Cordeiro L, Sarmento A. Seroprevalence of viral transfusion transmissible infections (HBsAg, anti-HCV, anti-HIV, Syphilis) and coinfection among healthy volunteer blood donors during 5-years in Luanda, Angola. Braz J Infect Dis. 2023;27:103704. https://doi.org/10.1016/j.bjid.2023.103704
10. Guimarães Nebenzahl H, Lopes A, Castro R, Pereira F. Prevalence of human immunodeficiency virus, hepatitis C virus, hepatitis B virus and syphilis among individuals attending anonymous testing for HIV in Luanda, Angola. S Afr Med J. 2013;103:186-188. https://www.scielo.org.za/pdf/samj/v103n3/30.pdf
11. Valente F, Lago BV, Castro CA, Almeida AJ, Gomes SA, Soares CC. Epidemiology and molecular characterization of hepatitis B virus in Luanda, Angola. Mem Inst Oswaldo Cruz. 2010;105:970-977. https://doi.org/10.1590/s0074-02762010000800004
12. Constantino AF, Proenca HM, Rodrigues T, Nicolau L, de Moura MC. Epidemiology of hepatitis B virus (HBV) infection in Luanda (Angola): prevalence and risk factors. Hepatology. 2009; 48(S1):751A.
13. Apata IW, Averhoff F, Pitman J, et al. Progress toward prevention of transfusion-transmitted hepatitis B and hepatitis C infection – sub-Saharan Africa, 2000–2011. MMWR Morb Mort Wkly Rev. 2014; 63:613-619. https://www.cdc.gov/MMWr/preview/mmwrhtml/mm6329a2.htm
14. Oliveira D, Martins MdR, Castro R, Cordeiro L, Barroso MR, Nazaré MA, Pereira F. Seropositivity rate and sociodemographic factors associated to HIV, HBV, HCV and syphilis among parturients from Irene Neto Maternity of Lubango City, Angola. Sex Transm Infect. 2020;96:587-589. https://doi.org/10.1136/sextrans-2019-054249
15. Peliganga LB, Horta MAP, Lewis-Ximenez LL. Enduring challenges despite progress in preventing mother-to-child transmission of hepatitis B virus in Angola. Pathogens. 2022;11:225. https://doi.org/10.7196/samj.6097
16. Steele AD, Bos P. Hepatitis B and C virus infection in adult volunteers in Angola. S Afr Med J. 1996;86:701-702.

17. Riches N, Henrion MYR, MacPherson P, et al. Vertical transmission of hepatitis B virus in the WHO African region: a systematic review and meta-analysis. Lancet Glob Health. 2025;13:3447-458. https://doi.org/10.1016/S2214-109X(24)00506-0

18. The State of the World's Children 2024: The Future of Childhood in a Changing World Statistical Compendium. New York: UNICEF; 2024. https://data.unicef.org/resources/sowc-2024/
