# Supplementary material for: Hepatitis B (HBsAg) prevalence among obstetric patients in Caluquembe, Angola, 2023–2024
Source: PLoS One. 2025 Jul 3;20(7):e0327426. doi: 10.1371/journal.pone.0327426 (PMC12225797; doi:10.1371/journal.pone.0327426)
Supplement: S3 File — (PDF) [file pone.0327426.s003.pdf]

Resumen en Español

Prevalencia de la hepatitis B (HBsAg) entre pacientes obstétricas en Caluquembe, Angola, 2023-2024

Objetivos: Los recién nacidos que contraen infecciones por el virus de la hepatitis B (VHB) al nacer a menudo desarrollan infecciones crónicas que pueden causar cirrosis, cáncer de hígado y la muerte en la edad adulta media. Las dosis al nacer de la vacuna contra la hepatitis B pueden salvar la vida de los bebés nacidos de madres con hepatitis B. Nuestro objetivo era medir la prevalencia del VHB entre las pacientes de maternidad en Huila, un distrito rural en el suroeste de Angola. Métodos: Se realizó un estudio de serie de casos prospectivo entre 317 mujeres peripartales en el Hospital Evangélico de Caluquembe desde noviembre de 2023 hasta febrero de 2024. Cada participante recibió una prueba en el lugar de atención del antígeno de superficie de la hepatitis B (HBsAg) y se le preguntó sobre el VHB y sus conocimientos sobre las vacunas. También realizamos entrevistas cualitativas sobre la prevención del VHB con 26 trabajadores de la salud. Resultados: La prevalencia de HBsAg fue de 4,7%. Ninguna de las mujeres que dieron positivo conocía previamente su estado. Sólo alrededor de un tercio de las mujeres estaban familiarizadas con el VHB o las vacunas contra la hepatitis B, y casi ninguno de los participantes informó que sus hijos mayores habían recibido la vacuna contra el VHB. Las trabajadores de salud materna propusieron organizar reuniones comunitarias para brindar educación sobre el VHB y la vacunación con dosis al nacer. Conclusiones: Solo aproximadamente la mitad de los bebés angoleños nacen en centros de salud, pero más del 80% de las mujeres asisten al menos a una consulta prenatal. Mejorar el acceso y la participación en la detección de la hepatitis B durante los controles prenatales es fundamental para garantizar que los bebés nacidos de mujeres con infecciones crónicas de hepatitis B puedan recibir la vacuna contra la hepatitis B al nacer.
