# Supplementary material for: Hepatitis B (HBsAg) prevalence among obstetric patients in Caluquembe, Angola, 2023–2024
Source: PLoS One. 2025 Jul 3;20(7):e0327426. doi: 10.1371/journal.pone.0327426 (PMC12225797; doi:10.1371/journal.pone.0327426)
Supplement: S4 File — (PDF) [file pone.0327426.s004.pdf]

Résumé en Français

Prévalence de l'hépatite B (AgHBs) chez les patientes en obstétrique à Caluquembe, Angola, 2023-2024

Objectifs : Les nouveau-nés infectés par le virus de l'hépatite B (VHB) à la naissance développent souvent des infections chroniques pouvant entraîner une cirrhose, un cancer du foie et le décès à l'âge adulte moyen. Les doses de vaccin contre l'hépatite B administrées à la naissance peuvent sauver la vie des bébés nés de mères infectées par le virus de l'hépatite B. Notre objectif était de mesurer la prévalence de l'hépatite B parmi les patientes en maternité à Huíla, une province rurale du sud-ouest de l'Angola.

Méthodes : Nous avons mené une étude prospective de série de cas auprès de 317 femmes en période périnatale à l'hôpital Evangélico de Caluquembe de novembre 2023 à février 2024. Chaque participante a subi un test de dépistage de l'antigène de surface de l'hépatite B (HBsAg) au point de service et a été interrogée sur ses connaissances concernant le VHB et les vaccins. Nous avons également mené des entretiens qualitatifs sur la prévention du VHB auprès de 26 professionnels de santé.

Résultats : La prévalence de l'HBsAg était de 4,7 %. Aucune des femmes testées positives n'était au courant de son statut. Seulement environ un tiers des femmes ont déclaré familiarité avec le VHB ou les vaccins contre l'hépatite B, et presque aucun n'a signalé que ses enfants plus âgés avaient reçu des vaccins contre le VHB. Les agents de santé maternelle ont proposé d'organiser des réunions communautaires pour sensibiliser les populations au VHB et à la vaccination à la naissance.

Conclusions : Seulement environ la moitié des bébés angolais naissent dans des établissements de santé, mais plus de 80 % des femmes participent à au moins une consultation prénatale.

Il est essentiel d’améliorer l’accès au dépistage de l’hépatite B pendant les soins prénatals pour garantir que les bébés nés de femmes atteintes d'hépatite B chronique reçoivent une dose de vaccin contre l'hépatite B à la naissance.
